# Anabolic Properties of High Mobility Group Box Protein-1 in Human Periodontal Ligament Cells *In Vitro*

**DOI:** 10.1155/2014/347585

**Published:** 2014-11-27

**Authors:** Michael Wolf, Stefan Lossdörfer, Piero Römer, Rogerio Bastos Craveiro, James Deschner, Andreas Jäger

**Affiliations:** ^1^Department of Orthodontics, University of Bonn, Welschnonnenstraße 17, 53111 Bonn, Germany; ^2^Department of Orthodontics, University of Regensburg, Franz-Josef-Strauss-Allee 11, 93053 Regensburg, Germany; ^3^Department of Pediatric Hematology and Oncology, Center for Pediatrics, University of Bonn Medical Center, Adenauerallee 119, 53113 Bonn, Germany; ^4^Experimental Dento-Maxillo-Facial Medicine, University of Bonn, Welschnonnenstraße 17, 53111 Bonn, Germany

## Abstract

High mobility group box protein-1 (HMGB1) is mainly recognized as a chemoattractant for macrophages in the initial phase of host response to pathogenic stimuli. However, recent findings provide evidence for anabolic properties in terms of enhanced proliferation, migration, and support of wound healing capacity of mesenchymal cells suggesting a dual role of the cytokine in the regulation of immune response and subsequent regenerative processes. Here, we examined potential anabolic effects of HMGB1 on human periodontal ligament (PDL) cells in the regulation of periodontal remodelling, for example, during orthodontic tooth movement. Preconfluent human PDL cells (hPDL) were exposed to HMGB1 protein and the influence on proliferation, migration, osteogenic differentiation, and biomineralization was determined by MTS assay, real time PCR, immunofluorescence cytochemistry, ELISA, and von Kossa staining. HMGB1 protein increased hPDL cell proliferation, migration, osteoblastic marker gene expression, and protein production as well as mineralized nodule formation significantly. The present findings support the dual character of HMGB1 with anabolic therapeutic potential that might support the reestablishment of the structural and functional integrity of the periodontium following periodontal trauma such as orthodontic tooth movement.

## 1. Introduction

In the course of orthodontic treatment, a remodeling process within the tooth supporting tissues including alveolar bone facilitates the movement and subsequent stabilization of teeth in a new position. The active phase of tooth movement is followed by an early and late phase of periodontal regeneration serving the reestablishment of the periodontal architecture and functional integrity. In the initial phase of host response to the mechanical stimulus, immune cells, mainly macrophages, are attracted to migrate to the stressed sites to clear necrotic cellular debris and prepare the local microenvironment for a subsequent regeneration of a functional periodontium by competent residual cells of the periodontal ligament (PDL) in the late phase [1]. PDL cells represent a mixed population of mainly fibroblastic phenotype, many of them exhibiting osteoblastic properties [2], but also host several stem and progenitor cells capable of forming all relevant periodontal tissues, including gingiva, the periodontal fibre apparatus, root cementum, and alveolar bone [3–7]. According to recent reports, PDL cells contribute to the regulation of the periodontal repair process by the release of specific mediators including proregenerative proteins and proinflammatory cytokines such as OPG, IGFs, IL6, IL8, TNF-alpha, and HMGB1 [4] in response to mechanical loading which then initiate an interaction with immune competent cells. Among those mediators, high mobility group box protein-1 (HMGB1) has gained scientific attention as an alarm in signaling tissue damage and has been characterized as a proinflammatory cytokine which is released by cells undergoing necrosis [8, 9]. In contrast to physiological conditions, where HMGB1 is primarily localized in the nucleus and regulates gene expression, necrotic stimuli cause the secretion and transition of the protein into the cytoplasm and further on into the extracellular space, where it was assigned multiple immunologic and osteometabolic functions [9]. Like the RANK/RANKL/OPG system, HMGB1 in cooperation with the circulating decoy receptor sRAGE mediates the interplay of immune cells and modifies the chemotaxis, proliferation, and expression of proinflammatory cytokines in target cells. HMGB1 initiates the physiologic remodeling process and is held accountable for the development of pathophysiologic tissue damage [10].

Within the bony microenvironment, HMGB1 was demonstrated to act as a chemoattractant for osteoblasts and osteoclasts during endochondral ossification as it does for monocytes and other immune and nonimmune competent cells [11].

It was shown that RAGE knockout mice display a higher bone density with, at the same time, reduced osteoclastic activity. Moreover, HMGB1 seems to mediate the RANKL-induced differentiation of osteoclastic precursors both* in vitro* and* in vivo*, as demonstrated in RANKL stimulated bone marrow macrophages of mice [12]. Finally, data from animal experiments demonstrated a distinct reduction of inflammatory host response upon inhibition of HMGB1 release by a specific antibody [13].

Little is known about the role of HMGB1 in the regulation of alveolar bone remodeling. Recently, a basal HMGB1 expression was demonstrated in PDL cells and a possible role in the regulation of alveolar bone resorption was derived from that observation. This conclusion was based on the finding that HMGB1 enhances the expression of proinflammatory and osteoclast inducing cytokines, such as IL-1*β*, Il-6, IL-17, and RANKL [14]. Finally, higher levels of HMGB1 were reported in the gingival crevicular fluid of patients suffering from periodontitis as compared to healthy individuals [15, 16].

Apart from its catabolic effect, potential anabolic properties of HMGB1 were deduced from experiments establishing the protein as a stimulating factor for wound healing and regeneration by enhancing cell proliferation and osteogenic differentiation [17–19].

Picking up on previous findings of our own group, where a sustained elevation of HMGB1 protein expression was reported in the late phase of periodontal repair in a rat model of orthodontic tooth movement [20], it was the aim of the present study to address HMGB1 induced changes of cell proliferation, migration, and osteogenic differentiation of human PDL cells* in vitro*. We hypothesized that HMGB1 protein would modulate those parameters such that a support of periodontal regeneration could be discussed.

## 2. Material and Methods

All experimental protocols were reviewed and approved by the Ethics Committee of the University of Bonn (reference number 029/08).

### 2.1. PDL Cell Culture

To avoid contamination with gingival or apical tissue, human PDL cells were explanted from the middle third of the tooth root from extracted premolars of three different adolescent donors (12–14 years of age) as described previously [21]. Extractions were performed for orthodontic reasons and with written parental consent.

Fifth passage, nonpredifferentiated PDL cells were seeded into 24-well plates (*n* = 6) in a density of 10000 cells/well and cultured to subconfluence (~70%) before starting the experiments. This setup was used for the immunohistochemistry, mineralization assays, ALP, and osteocalcin protein analyses. Deviating seeding densities are mentioned below. Cells were cultured in DMEM containing 10% fetal bovine serum and 0.5% antibiotics (diluted from a stock solution containing 5000 U/mL penicillin and 5000 U/mL streptomycin; Biochrom AG, Germany) at 37°C in an atmosphere of 100% humidity, 95% air, and 5% CO_2_. Prior to experimental use, cells were characterized for their mesenchymal origin as described previously [22].

### 2.2. PDL Cell Proliferation Experiments

To investigate the effect of HMGB1 on PDL cell proliferation, human PDL cells were stimulated with 50 ng/mL HMGB1 protein (GenWay-Biotech, USA) according to the previously published protocol for 3 days [17]. Vehicle-treated (H_2_O) PDL cells served as controls. A MTS assay was performed to measure the cell proliferation rate according to the manufacturer's instructions (Promega, Germany). In this assay, PDL cells were seeded in a density of 2000 cells/well. Since those experiments did not reveal any concentration dependence between the tested amount of 50 ng/mL and 100 ng/mL HMGB1 protein, all of the following experiments were performed with 50 ng/mL HMGB1 only, according to the protocol of Meng et al. [17].

### 2.3. PDL Cell Migration Assay

To address the influence of HMGB1 on PDL cell migration, transwell assays were performed. Briefly, the lower chamber of 24-transwell-plates (Corning, USA) was filled with either culture medium for control purposes or with conditioned medium containing 50 ng/mL recombinant HMGB1 protein, respectively. Carboxyfluorescein diacetate succinimidyl ester-labeled PDL cells were cultured in a density of 10000 cells/well in the upper chamber. After 3 h of incubation, the number of cells migrated to the bottom of the migration filter in the lower chamber was determined using fluorescence imaging according to a modification of a previously established protocol [23]. To quantify the amount of migrated PDL cells, images of the migration filter were assessed semiquantitatively using a range starting from 0 (meaning no cell migration visible) to 1 (meaning the total bottom of the filter was covered by cells). Migrated PDL cells were assessed in 0.1 steps.

### 2.4. Immunocytochemistry

To demonstrate the effect of HMGB1 on early and late osteoblastic differentiation of hPDL cells, immunocytochemical staining on cover was performed using either a 1 : 50 (40 *μ*g/mL) working dilution of the primary antibody for ALP (rabbit anti-human; Quartett, Germany) or 1 : 25 (80 *μ*g/mL) for osteocalcin (rabbit anti-human; Biozol, Germany) in combination with a secondary fluorescence-labeled (Cy-3) antibody (1 : 100 (1.5 *μ*g/mL) working dilution; goat anti-rabbit; Invitrogen, Germany) and analyzed microscopically as reported previously [24]. Negative control were run by (i) omitting the primary antibody, (ii) omitting the primary and secondary antibody and using TBS/BSA instead, and (iii) by substituting the primary antibody by an unspecific IgG.

### 2.5. Gene Expression Experiments and Real Time PCR

To determine the effect of HMGB1 on mRNA expression of osteogenic marker genes with an expected peak occurring earlier at the transcriptional level than at the protein level, fifth passage PDL cells seeded in a density of 30000 cells/well in 6 wells plates using the protocol as mentioned above. At subconfluence PDL cells were exposed to 50 ng/mL HMGB1 protein for 3 h. Vehicle-treated cultures served as controls. At harvest, the expression of candidate genes associated with osteogenic differentiation including alkaline phosphatase, osteopontin, osteocalcin, RUNX2, bone morphogenetic proteins 2 and 4 (BMP-2, BMP-4), and cementum protein-1 (CEMP1) was analyzed by real time PCR as described previously [25]. The primer sequences used were as follows: ALP sense 5′-AGA-GAA-AGC-GAT-GGT-GGA-TG-3′, antisense 5′-CGG-TGG-CAT-TAA-TAG-TGA-GAT-G-3′ [26]; CEMP1 sense 5′-CCC-ACA-CCT-CAA-AAT-CAT-CC-3′, antisense 5′-CAG-GGT-CAG-GGT-GAG-AGA-GA-3′ (Roche Diagnostics); osteopontin sense 5′-AGG-AGG-AGG-CAG-AGC-ACA-3′, antisense 5′-CTG-GTA-TGG-CAC-AGG-TGA-TG-3′ [27]; osteocalcin sense 5′-ATG-AGA-GCC-CTC-ACA-CTC-CTC-G-3′, antisense 5′-GTC-AGC-CAA-CTC-GTC-ACA-GTC-C-3′; RUNX2 sense 5′-ATG-GCA-TCA-AAC-AGC-CTC-TTC-AGC-A-3′, antisense 5′-CGT-GGG-TTC-TGA-GGC-GGG-ACAC-C-3′; BMP-2 sense 5′-CTC-GGC-CTT-GCC-CGA-CAC-TGA-3′, antisense 5′-TAA-GAA-GCA-CGC-GGG-GAC-ACG-T-3′; BMP-4 sense 5′-CCT-GGT-AAC-CGA-ATG-CTG-ATG-GTC-G-3′, antisense 5′-AGA-CTG-AAG-CCG-GTA-AAG-ATC-CCG-C-3′. *β*-actin was used as an endogenous reference (sense 5′-CAT-GGA-TGA-TGA-TAT-CGC-CGC-G-3′, antisense 5′-ACA-TGA-TCT-GGG-TCA-TCT-TCT-CG-3′) [26].

### 2.6. ALP Protein Expression

To analyze the HMGB1 effect on early differentiation of hPDL cells along the osteoblastic pathway, fifth passage cells were either cultured in the presence of 50 ng/mL HMGB1 protein for 48 h or of the respective vehicle to serve control purposes. At harvest, cells were released from the culture surface by trypsinization for 10 min at 37°C. This reaction was terminated by the addition of DMEM containing 10% FBS. Thereafter, the cell suspension was centrifuged and the cell pellet resuspended in 0.01% TritonX-100. Changes in ALP specific activity were measured at the protein level in lysates of isolated cells as a function of release of paranitrophenol from paranitrophenylphosphate at pH 10.2 as described previously [28]. To assure that changes in ALP activity do not simply result from changes in proliferation or apoptosis rather than from direct HMGB1 effects, ALP data was calculated as a function of the protein content of the sample [25].

### 2.7. Osteocalcin Protein Expression

The level of osteocalcin in the conditioned media was assayed using a commercially available enzyme-linked immunoassay (ELISA) kit specific for human osteocalcin (IBL GmbH, Germany) according to the manufacturer's instructions.

### 2.8. Mineralization Assay

To analyze the effect of HMGB1 on PDL cell differentiation capacity, PDL cells were cultured on cover slips for 21d in osteogenic medium (OM) containing 10^−12^ M *β*-glycerolphosphate and 82 mg/L ascorbic acid and OM supplemented with 50 ng/mL HMGB1, respectively. For positive control, 10^−7^ M dexamethasone was supplemented to the OM medium. For negative control, PDL cells were cultured in cell culture medium as described above. After 21d of culture, cells were further processed for visualization of mineralization by von Kossa staining and subsequent histomorphometric evaluation.

### 2.9. von Kossa Staining

Cells were fixed with 4% paraformaldehyde in PBS for 10 min. After washing, cells were incubated with 5% silver nitrate solution at 4°C for 40 min in the dark and neutralized in 1% pyrogallol for 5 min. Cells were fixed with 5% sodium thiosulfate for 5 min and counterstained with 0.1% nuclear fast red in 5% aluminum sulfate for 10 min. The cover slips were dehydrated in 100% ethanol and xylol before slides were cover slipped with DePex.

Two images were captured per well at ×10 magnification and the mineralized area was quantified as a function of total area. The data was analyzed by analysis of variance (ANOVA) and Bonferroni's modification of student's *t*-test for multiple comparisons.

### 2.10. Statistical Analysis

All data were analyzed by analysis of student's *t*-test, variance (ANOVA), and Bonferroni's modification of student's *t*-test for multiple comparisons or Wilcoxon test. *P* values <0.05 were considered to be significant. The data are representative of two replicate experiments, which both yielded similar results.

## 3. Results

### 3.1. HMGB1 Effect on PDL Cell Proliferation

**Figure 1:**
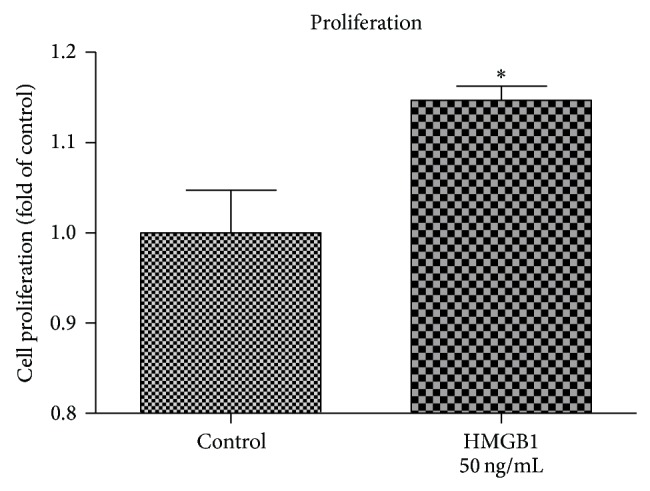
HMGB1 enhances PDL cell proliferation. As determined by MTS assay, HMGB1 stimulation resulted in an increase in hPDL cells proliferation. Data represent the mean ± SD for six independent cultures. ^*^ *P* < 0.05, experimental group versus untreated control.

A 72 h exposure of hPDL cells to HMGB1 resulted in a significant increase of PDL cell proliferation. As compared to untreated controls, in HMGB1 treated human PDL cells a ~17% increase in proliferation was observed (Figure 1).

### 3.2. Influence of HMGB1 on PDL Cell Migration

**Figure 2:**
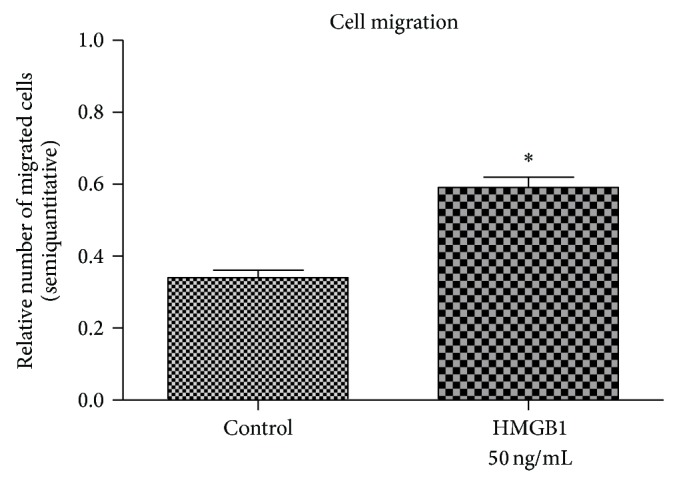
HMGB1 stimulates PDL cell migration. HMBG1 treatment of hPDL cultures resulted in a significantly higher number of cells migrating from the upper to the lower chamber of the transwell assay system. Data represent the mean ± SD for six independent cultures. ^*^ *P* < 0.05, experimental group versus untreated control.

HMGB1 challenge led to an increased migration of hPDL cells through the transwell filter. Compared to untreated controls, the addition of experimental media supplemented with HMGB1 approximately doubled migration of PDL cells from the upper chamber (Figure 2).

### 3.3. Gene Expression

**Figure 3:**
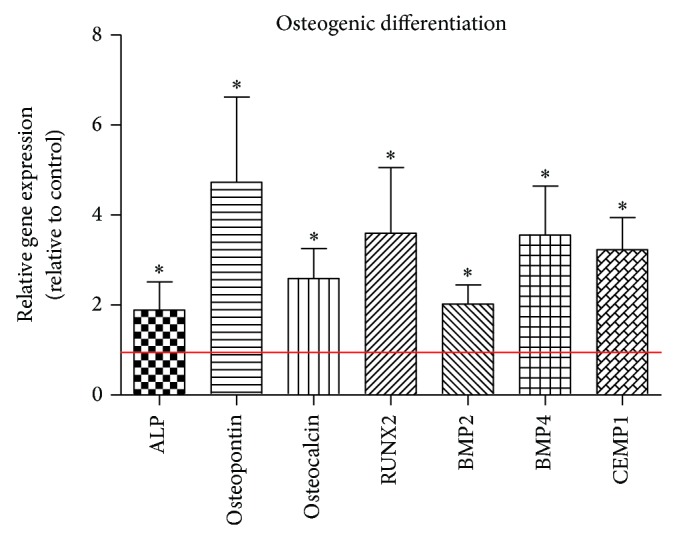
HMGB1 stimulates PDL cell osteogenic differentiation parameters. Osteogenic differentiation parameters were stimulated significantly at the transcriptional level in hPDL cells as determined by real time PCR. Data represent the mean ± SD for six independent cultures. ^*^ *P* < 0.05, experimental group versus untreated control (red line).

Differentiation experiments demonstrated a significant impact of HMGB1 on the respective parameters in hPDL cells. Following a stimulation with 50 ng/mL HMGB1, a significantly increased gene expression was observed for ALP, BMP-2, BMP-4, osteopontin, osteocalcin, RUNX2, and cementum protein-1 (Figure 3).

### 3.4. HMGB1 Effect on Osteogenic Differentiation

**Figure 4:**
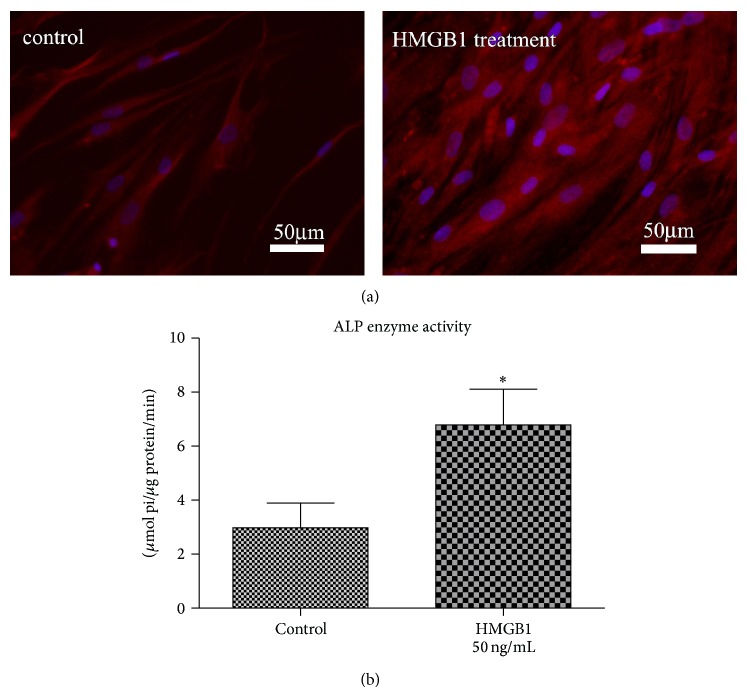
HMGB1 increases ALP protein expression. In addition to the transcriptional level, alkaline phosphatase specific activity was also enhanced at the protein level as visualized by immunofluorescent staining of hPDL cells (a). The visual impression was further confirmed and quantified by biochemical assay (b). Data represent the mean ± SD for six independent cultures. ^*^ *P* < 0.05, experimental group versus untreated control.

**Figure 5:**
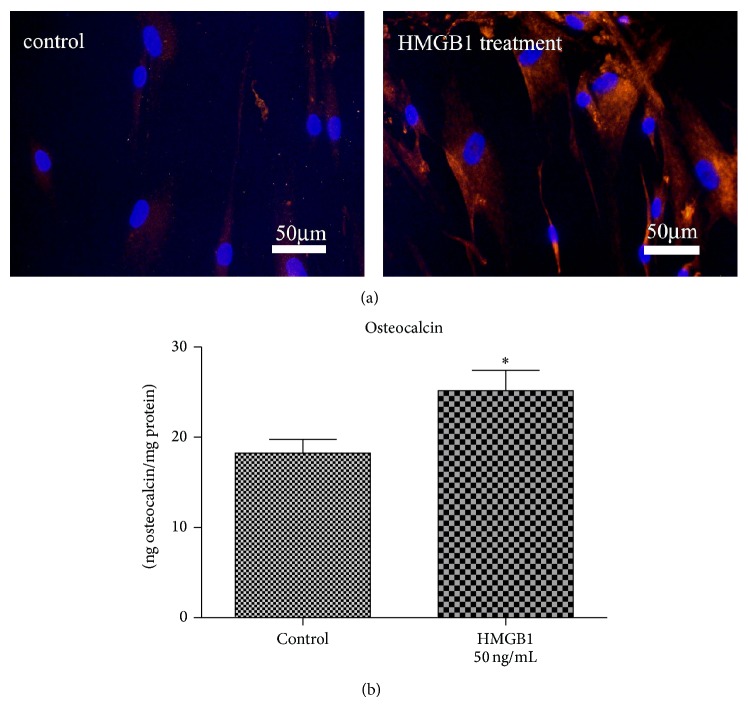
HMGB1 upregulates osteocalcin protein expression. In addition later stages of maturation were also affected by HMGB1. Enhanced osteocalcin protein expression was demonstrated, both in immunofluorescent stainings and by osteocalcin ELISA. Data represent the mean ± SD for six independent cultures. ^*^ *P* < 0.05, experimental group versus untreated control.

The analysis of HMGB1 for its capacity to modulate the osteogenic differentiation of hPDL cells revealed an upregulation of ALP specific activity in response to HMGB1 by approximately factor 2.2 (Figures 4(a) and 4(b)). Similar data were obtained for osteocalcin protein expression, although the HMGB1 effect was less pronounced regarding this parameter but was nevertheless still significant (Figures 5(a) and 5(b)).

### 3.5. PDL Cell Mineralization

**Figure 6:**
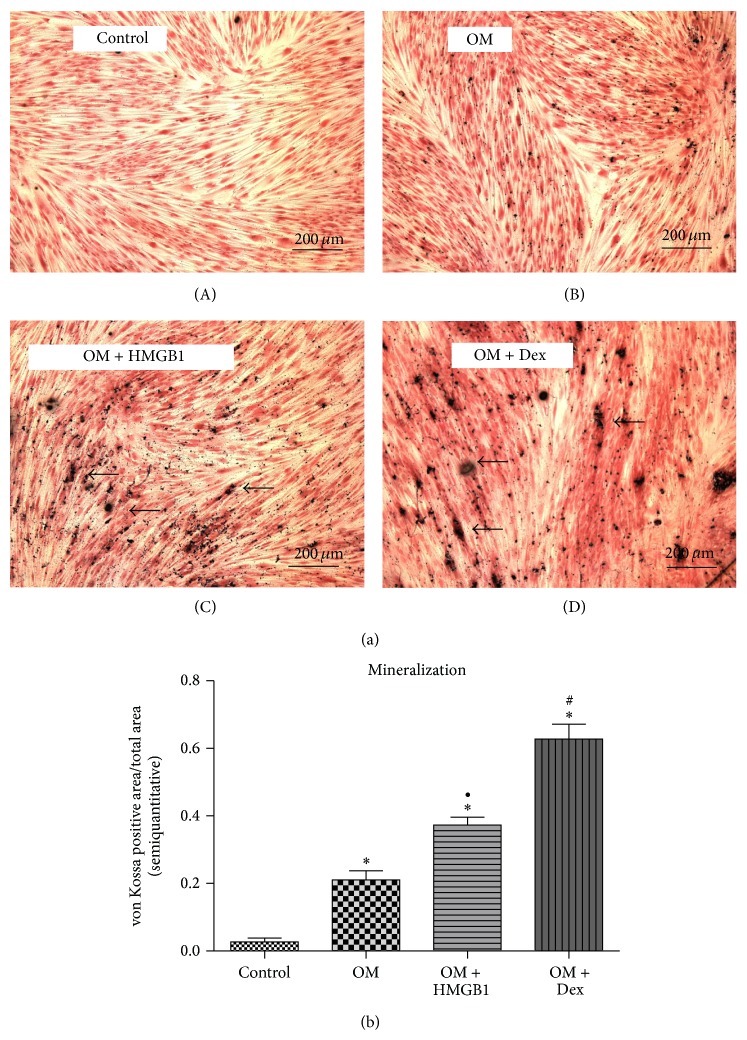
HMGB1 stimulation influences PDL cell mineralization. HMGB1 exposure of hPDL cells for 21d resulted in enhanced biomineralization (a). In the absence of a respective substrate, no mineralized nodule formation was observed (A), whereas osteogenic medium (OM) itself (B) as well as OM supplemented with HMGB1 (C) led to the formation silver nitrate positive areas (arrows), which were most evident and spread in dexamethasone treated cultures (D). The semiquantitative assessment of mineralized area versus total area revealed confirming results (b). Data represent the mean ± SD for six independent cultures. ^*^ *P* < 0.05, experimental group versus untreated control; ^#^ *P* < 0.05, dexamethasone treated group versus HMGB1 treated cultures; ^•^ *P* < 0.05, HMGB1 treated cultures versus osteogenic medium alone.

In a 21d stimulation experiment, HMGB1 treatment turned out to affect biomineralization as well. As expected, PDL cells cultured in the absence of the substrate *β*-glycerolphosphate did not show any mineralized nodule formation. In comparison to *β*-glycerolphosphate stimulation alone, the addition of 50 ng/mL HMGB1 led to a further significant increase of silver nitrate enriched areas which, regarding the order of magnitude, leveled in the middle between the effect seen for OM alone and dexamethasone treated cultures, the latter showing the expected highest rate of mineralization after 21d (Figure 6(a)). This visual impression was further confirmed by the semiquantitative assessment of von Kossa positive areas (Figure 6(b)).

## 4. Discussion

The present study examined potential anabolic effects of HMGB1 on human PDL cells which could possibly modify and support regenerative periodontal processes, for example, following orthodontic tooth movement. There is considerable conclusiveness among our data that HMGB1 increases proliferation, migration, osteogenic differentiation both, at the transcriptional and translational level, and extracellular matrix biomineralization.

Apart from the well-established role of HMGB1 as a chemoattractant and catabolic protein for macrophages in inflammatory periodontal disease or in the initial phase of host response to local stress resulting in an increased migration of immune cells in the local defect surroundings [29, 30] and an enhanced release of other inflammatory mediators into the local microenvironment [8, 9, 31], recent reports suggest anabolic properties of HMGB1 within the subsequent phase of tissue regeneration as well. According to data collected in a cardiologic infarction mouse model, HMGB1 was demonstrated to act particularly on pluripotent stem and progenitor cells streaming in the defect surroundings and, thereby, promote the regeneration efficiency [32]. In a different experimental approach where human cardiac fibroblasts were stimulated with HMGB1, Rossini et al. demonstrated increased levels of growth factors including vascular endothelial growth factor, basic fibroblast growth factor, and insulin-like growth factor-1 [33]. Further support is added by experiments in a diabetic mouse wound model, where HMGB1 promoted wound closure, granulation tissue formation, and angiogenesis, eventually enhancing wound healing [34]. In a similar experimental study using 3T3 fibroblasts, HMGB1 was reported to stimulate wound healing by enhancing cell proliferation and migration [35]. It should not remain unmentioned that conflicting reports also exist in this context. Some studies suggest an inhibitory role of HMGB1 in wound healing resulting from an impairment of collagen synthesis along with an upregulation of collagen degradation and expression of matrix metalloproteinase genes 8 and 9, which could be neutralized by ethyl pyruvate (EP), a potent inhibitor of HMGB1 release [36, 37], resulting in enhanced collagen deposition and tensile wound strength in the EP treated specimens. However, despite these apparently conflicting results, HMGB1 seems to occupy a central role in the mediation of a local and systemic response to diverse stimuli and might bear diagnostic and therapeutic potential. The suitability of the HMGB1/sRAGE ratio as a diagnostic tool giving evidence for disease progression has been proven in multiple sclerosis patients, where serum and cerebral fluid measurements indicated an increase of the ratio, resulting from reduced sRAGE levels in diseased patients as compared to healthy control individuals [38]. Likewise, in tumor patients an increased pathogenicity could be correlated to reduced serum levels of sRAGE as an indirect hint for a proportionately enhanced HMGB1 expression [39].

Meng and coworkers showed HMGB1 to promote the migration of mesenchymal stem cells and their differentiation along the osteoblastic pathway and concluded an important role for HMGB1 in bone restoration [17]. In our experiments, PDL cell proliferation, migration, and osteogenic differentiation were increased by HMGB1 as well, the latter being more pronounced regarding early osteoblastic maturation stages, as evidenced by a more pronounced increase of alkaline phosphatase specific activity and osteopontin expression in comparison to osteocalcin production. In the light of these considerations, it seems reasonable to conclude a similar supportive role for HMGB1 in periodontal regeneration, since the PDL hosts cells at all different developmental stages ranging from stem cells to terminally differentiated fibroblasts, osteoblasts, and so forth. Likewise, the stimulation of extracellular matrix mineralization by HMGB1 is an important finding, since this parameter is crucial in the accomplishment of periodontal regeneration. Combining the present data with literature reports, it is likely that multiple mechanisms are involved in the HMGB1 driven control of innate immune response and repair of tissue damage, ranging from a direct stimulation of PDL fibroblasts and more indirect signaling affecting angiogenesis and macrophage physiology [19]. Collectively, our findings strengthen the idea of a three-legged action of HMGB1, with nuclear HMGB1 maintaining chromatin architecture and allowing for undisturbed transcription activities, extracellular HMGB1 alerting the innate immune system to tissue damage and mediating inflammation [17], but also driving tissue remodeling towards regeneration once the central inflammatory response of the local tissues has been overcome. Future experiments analysing the interaction of HMGB1, PDL cells, and osteoblasts in coculture models with special regard to its ability to modify extracellular matrix synthesis are needed to gain further insights into how HMGB1 protein functions in periodontal remodelling.

In summary, the present data add further support to the idea that hPDL cells contribute to the orchestration of periodontal remodelling and regeneration, for example, in the course of orthodontic tooth movement. The findings corroborate the idea of a dual role for HMGB1 with both, catabolic and anabolic properties, the latter being demonstrated here by HMGB1-induced enhanced proliferation, migration, osteoblastic differentiation, and biomineralization of hPDL cells. Those findings add another piece to the mosaic for a better understanding of the cellular regulatory mechanisms underlying periodontal remodeling. A broad scientific basis is the crucial prerequisite for the future development of immune based clinical applications in the attempt to better monitor periodontal tissue reactions to mechanical intervention, support periodontal regenerative processes, and reduce the risk of unwanted side effects during orthodontic treatment.
